# Halichoblelide D, a New Elaiophylin Derivative with Potent Cytotoxic Activity from Mangrove-Derived *Streptomyces* sp. 219807

**DOI:** 10.3390/molecules21080970

**Published:** 2016-07-25

**Authors:** Ying Han, Erli Tian, Dongbo Xu, Min Ma, Zixin Deng, Kui Hong

**Affiliations:** Key Laboratory of Combinatorial Biosynthesis and Drug Discovery, Ministry of Education, School of Pharmaceutical Sciences, Wuhan University, Wuhan 430071, China; hanying0928@whu.edu.cn (Y.H.); 2014103060011@whu.edu.cn (E.T.); xudongbo@whu.edu.cn (D.X.); winder_568@163.com (M.M.); zxdeng@whu.edu.cn (Z.D.)

**Keywords:** mangrove actinomycetes, *Streptomyces* sp. 219807, elaiophylin, cytotoxic

## Abstract

During our search for interesting bioactive secondary metabolites from mangrove actinomycetes, the strain *Streptomyces* sp. 219807 which produced a high elaiophylin yield of 4486 mg/L was obtained. A new elaiophylin derivative, halichoblelide D (**1**), along with seven known analogues **2**–**8** was isolated and identified from the culture broth. Their chemical structures were determined by detailed analysis of 1D and 2D NMR and HRMS data. The absolute configuration of halichoblelide D (**1**) was confirmed by comparing the CD spectrum with those of the reported analogues. Compounds **1**–**7** exhibited potent cytotoxic activities against HeLa and MCF-7 cells with IC_50_ values ranging from 0.19 to 2.12 μM.

## 1. Introduction

There is a continuous and urgent demand for iterative cycles of drug discovery and development to combat new emerging human diseases and multi-drug resistant pathogens [1,2]. Microbial natural products have delivered a plethora of structurally diverse specialized metabolites with potent pharmaceutical properties [3]. Microorganisms derived from underexplored habitats, such as mangrove ecosystems, are especially noteworthy, in part owing to their unique biosynthetic potential and the corresponding attenuation of the likelihood of finding high numbers of known compounds [4,5,6]. Mangrove ecosystems are extensively distributed in the interface between terrestrial and marine environments at tropical and subtropical latitudes, thereby suffering from extremely harsh stresses, including temperature, salinity, moisture and anoxia [7]. Therefore, mangrove-derived actinomycetes are distinct from terrestrial isolates in physiology and genotype to cope with the rigorous environments. Several decades of studies have demonstrated that actinomycetes recovered from mangrove systems are increasingly recognized as a vast reservoir of new bioactive natural products, such as salinosporamide A [8], xiamycins [9], and streptocarbazoles A and B [10]. Accordingly, our studies are focused on the mangrove ecosystem actinomycetes.

**Figure 1:**
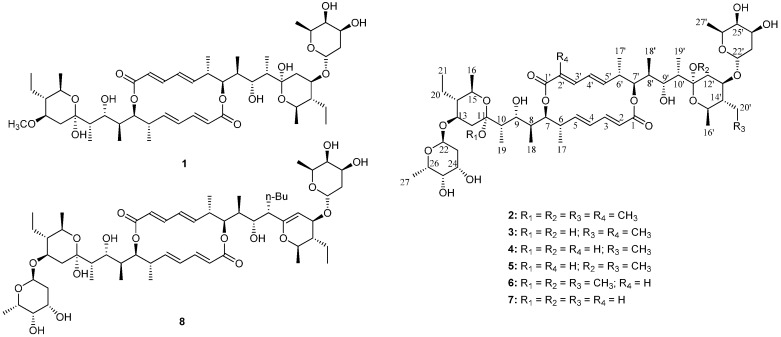
Chemical structures of compounds **1**–**8**.

After an initial cytotoxic screening of fermentation extracts of actinomycetes isolated from mangrove soil collected in Sanya, the active strain *Streptomyces* sp. 219807 was selected for further investigation. By means of HRMS match in the AntiBase 2012, elaiophylin and many unassigned elaiophylin-related compounds were successfully detected in the ethyl acetate extract of the fermentation broth. Elaiophylins are a family of glycosylated 16-membered macrolides featuring C2 symmetry produced by several *Streptomyces* spp. [11,12,13,14,15], which exhibit remarkable biological properties, such as antibacterial [16,17], anthelminthic [18], anticancer [19], antiviral [20] and immunosuppressant activities [21]. Chemical investigation of the bioactive extract led to the discovery of eight elaiophylins including a new derivative, halichoblelide D (**1**), together with 2-methyl-11,11′-*O*-dimethylelaiophylin (**2**), 2-methylelaiophylin (**3**), elaiophylin (**4**), 11-*O*-methyl-elaiophylin (**5**), 11,11′-*O*-dimethylelaiophylin (**6**), efomycin G (**7**) and 11′,12′-dehydroelaiophylin (**8**) (Figure 1). Details of their isolation, structure elucidation and bioactivities are reported herein.

## 2. Results

### 2.1. Structure Elucidation

**Figure 2:**
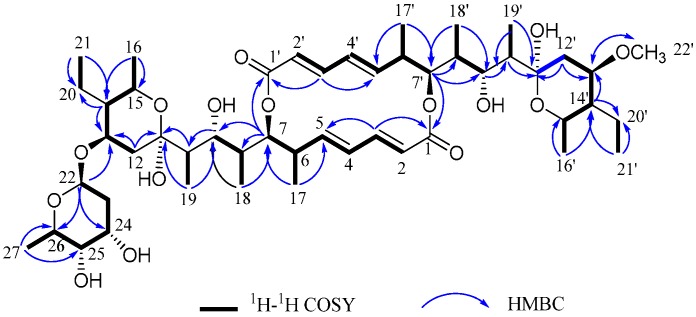
Selected HMBC and COSY correlations of compound **1** in CDCl_3_.

Halichoblelide D (**1**) was obtained as a white needle-like crystals. Its molecular formula was determined as C_49_H_80_O_15_ by the negative HRESIMS at *m*/*z* 943.5183 [M + Cl]^−^ (calc. for C_49_H_80_O_15_Cl^−^, 943.5186), implying 10 degrees of unsaturation. The UV (252 nm) and IR (1699, 1636 cm^−1^) absorption indicated the presence of an aliphatic conjugated ester system in the molecule [22]. A close inspection of the ^1^H-NMR (Table 1) and ^1^H-^1^H COSY spectra of **1** revealed the presence of two same sets of conjugated *trans*-dienes at δ_H_ 5.69 (1H, d, *J =* 15.4 Hz, H-2, H-2′), 6.99 (1H, dd, *J =* 15.3, 11.2 Hz, H-3, H-3′), 6.14 (1H, dd, *J =* 15.1, 11.3 Hz, H-4, H-4′), 5.64 (1H, dd, *J =* 15.0, 9.6 Hz, H-5, H-5′), together with a methoxy moiety, eleven methyl groups, thirty sp^3^-hybridized methines and methylenes. The ^13^C-NMR and DEPT spectra illustrated characteristic signals attributable to two carbonyls at δ_C_ 170.3, 170.2, two same groups of olefinic methines at δ_C_ 121.2, 145.3, 132.3, 144.5, along with two hemiketal carbon at δ_C_ 99.3, 99.4. Through detailed analysis of NMR data, the fragments of **1** resembled those of the known compound elaiophylin (**4**) (please see Supplementary Materials Table S1 for the detailed data) in the macrodiolide ring, polyketide side chains, hemiketal rings and one sugar moiety, except that the 2-deoxy-l-fucose sugar unit in **4** was replaced with a methoxyl group at δ_H_ 3.34 (3H, s)/δ_C_ 56.7. The methoxyl group was attached to C-13′ based on the HMBC correlation (Figure 2), and thus the planar structure of **1** was identified.

The measured vicinal coupling constants of the protons and NOESY correlations (Figure S6) are observed to be closely resembled those of the known halichoblelide B [23], allowing the assignment of the relative stereochemistry of **1**. Furthermore, the absolute configuration of **1** was concluded to be the same with halichoblelide B due to the nearly superimposable Cotton effects [23]. Therefore, the structure of compound **1** was determined as 13′-de-2-deoxy-l-fucose-13′-O-methylelaiophylin (Figure 2) and named as halichoblelide D following the examples of halichoblelide B and C [23].

Seven known metabolites were also isolated in this investigation and respectively identified as 2-methyl-11,11′-*O*-dimethylelaiophylin (**2**) [24], 2-methylelaiophylin (**3**) [24], elaiophylin (**4**) [11,19], 11-*O*-methylelaiophylin (**5**) [16], 11,11′-*O*-dimethylelaiophylin (**6**) [16], efomycin G (**7**) [14] and 11′,12′-dehydroelaiophylin (**8**) [17] by comparing their spectral data with the reported literature.

### 2.2. Yield of Elaiophylin in Shake-Flask Fermentation under Laboratory Conditions

Strain 219807 has been cultured on 18 different media available in our lab, and was found to produce the highest yield of elaiophylin on DO medium (50 mL in 250 mL Erlenmeyer flask), the yield of elaiophylin (**4**) measured by HPLC was calculated based on its standard curve [25]. The regression equation of elaiophylin was obtained as y = 630x − 1.6007, and the calibration curve showed good linearity (R^2^ = 0.9993) (Figure S11). According to the equation, the yield of elaiophylin reached 4486 mg/L in shake-flask fermentation under laboratory conditions, which was significantly more (>2 fold) than the highest yield reported before [26]. This high yield was primarily dependent on the strain of the microorganism. The proper medium DO which contained a complex carbon sources as observed by other reports [27,28] also contributed to the high production.

### 2.3. Cytotoxic Activity of Compounds ***1**–**7***

Compounds **1**–**7** showed potent activities against human cervical carcinoma HeLa and breast cancer MCF-7 cell lines with IC_50_ values ranging from 0.19 to 2.12 μM (Table 2). Remarkably, the new compound **1** exhibited cytotoxic activities against HeLa and MCF-7 cell lines with IC_50_ values of 0.30 and 0.33 μM, respectively. Compound **8** was not evaluated in cytotoxicity assays for the scarcity of material.

## 3. Experimental Section

### 3.1. General Procedures

Optical rotations were measured using a 341 polarimeter (Perkin-Elmer, Norwalk, CT, USA). UV spectra were recorded on a UV-2600 UV-VIS spectrophotometer (Shimadzu, Tokyo, Japan). IR spectra were obtained from Nicolet Nexus 470 FT-IR spectrometer (Thermo Fisher Scientific, Waltham, MA, USA). 1D and 2D NMR spectra were determined with AM-400 and DRX-500 spectrometers (Bruker, Ettlingen, Germany) using TMS as the internal standard. Circular dichroism spectra were recorded with a Chirascan spectropolarimeter (Applied Photophysics, Leatherhead, UK). High-resolution mass spectrometric (HRMS) data were measured on a linear trapquadrupole (LTQ)-Orbitrap Velos instrument (Thermo Fisher Scientific). Analytical HPLC was carried out on a Waters 2998 (Waters, Milford, MA, USA) using a Phenomenex Gemini C18 column (250 mm × 4.6 mm, 5 μm; Phenomenex, Torrance, CA, USA). The semi-preparative HPLC instrument used (Agilent 1260 Infinity, Agilent Technologies, Santa Clara, CA, USA) was equipped with an Agilent Zorbax SB-C18 column (250 mm × 9.4 mm, 5 μm). Thin-layer chromatography was conducted on precoated silica gel 60 GF254 plates (Qingdao Haiyang Chemical, Qingdao, China) and column chromatography was performed using silica gel (200–300 mesh; Qingdao Haiyang Chemical). 3-(4,5-dimethylthiazol-z-yl)-2,5-diphenyltetrazolium bromide (Amresco, Cleveland, OH, USA) and 5-fluorouracil (Aladdin, Shanghai, China) were employed for cytotoxicity assays. An Infinite M200 Pro microplate reader (Tecan, Mannedorf, Switzerland) was used for measuring the absorbance.

### 3.2. Material and Fermentation

The strain 219807 was isolated from mangrove soil collected in Sanya, Hainan Province, China and characterized as *Streptomyces* sp. by 16S rRNA sequence (GenBank accession number HQ992731). A voucher specimen was deposited in the Key Laboratory of Combinatorial Biosynthesis and Drug Discovery (Wuhan University, Wuhan, China), Ministry of Education, and Wuhan University School of Pharmaceutical Sciences (http://pharmacy.whu.edu.cn/NewsDetail.asp?MaxSort=xygk&MaxUrl=about&id=450) and the China Center for Type Culture Collection (CCTCC No：M 2015276).

The strain was pre-cultured on ISP2 agar medium (0.4% glucose, 0.4% yeast extract, 1% malt extract, pH 7.0, supplemented with 2% agar) for 5–7 days. A single colony was inoculated into 50 mL of ISP2 liquid medium in 250 mL Erlenmeyer flasks on a rotatory shaker (220 rpm) at 28 °C for 3 days. Subsequently, 10 mL of seed culture was inoculated to 1 L Erlenmeyer flask containing 250 mL of DO fermentation medium (1% glucose, 2.5% dextrin, 2% oatmeal, 1% cottonseed flour, 0.5% fish meal, 0.2% yeast extract, 0.3% CaCO_3_, pH 6.0). Total 5 L of the fermentation broth was incubated for 8 days at 220 rpm and 28 °C. The highest yield of elaiophylin (**4**) was obtained while using 50 mL liquid culture of DO medium in 250 mL flask, observed by HPLC and calculated based on its standard curve [25].

### 3.3. Extraction and Isolation

The fermentation broth was separated into the mycelium and supernatant after centrifuging at 12,000× *g* for 1 h at 4 °C. The mycelium was disrupted by 80% acetone aqueous solution through ultrasonication for three times and evaporated under reduced pressure to yield 10.0 g of crude extract. The extract was subjected to silica gel column chromatography using gradient elution with cyclohexane and acetone mixture (*v*/*v*, 90:10–0:100) to afford nine fractions: A1–A9. Fraction A5 was purified by reversed-phase HPLC (3 mL/min, UV detection at 252 nm) with a gradient of CH_3_CN (A)/H_2_O (B) (0–5 min, 65% A; 5–6 min, 65%–80% A; 6–14 min, 80% A; 14–15 min, 80%–95% A; 15–19 min, 95% A; 19–20 min, 95%–100% A; 20–23 min, 100% A; 23–25 min, 65% A), to yield **1** (2.8 mg, *t_R_* 23.6 min). Fraction A7 was purified by reversed-phase HPLC with a gradient of CH_3_CN (A)/H_2_O (B) (0–12 min, 79% A; 12–13 min, 79%–90% A; 13–29 min, 90% A; 29–30 min, 90%–79% A; 30–38 min, 79% A) to afford six subfractions A7A–A7F. Pure compounds **4** (75.3 mg, *t_R_* 5.5 min), **5** (170.2 mg, *t_R_* 14.1 min) and **6** (386.2 mg, *t_R_* 34.0 min) were directly obtained from A7A, A7B and A7E, respectively. Subfraction A7C was further purified by reverse-phase HPLC eluted with CH_3_CN/H_2_O (64:36) to give **8** (2.8 mg, *t_R_* 10.0 min). Subfraction A7D was separated by reverse-phase HPLC with an isocratic solvent system of CH_3_CN/H_2_O (53:47) to obtain **7** (15.2 mg, *t_R_* 10.5 min). Subfraction A7F was subjected to reversed-phase HPLC with a gradient of CH_3_CN (A)/H_2_O (B) (0–4 min, 80% A; 4–5 min, 80%–87% A; 5–12 min, 87% A; 12–13 min, 87%–99% A; 13–25 min, 99% A; 25–26 min, 99%–80% A; 26–30 min, 80% A) to afford **3** (5.1 mg, *t_R_* 13.1 min) and **2** (15.2 mg, *t_R_* 28.4 min).

*13′-De-2-deoxy-l-fucose-13′-O-methylelaiophylin* (*Halichoblelide D,* **1**): White needle-like crystals (2.8 mg), [α]${}_{D}^{20}$ −3.36 (c 0.055, MeOH); UV (MeOH) λmax (log ε) 252 (4.29) nm; IR (ν_max_ cm^−1^) 3523, 3445, 3333, 2977, 2931, 1699, 1636, 1458, 1382, 1302, 1222, 1184, 1088, 981, 807, 743, 695, 641, 567; CD (MeOH) 213 (Δε −14.2), 250 (Δε −34.9), 279 (Δε +23.9); HRESIMS: *m*/*z* 943.5183 [M + Cl]^−^ (calc. for C_49_H_80_O_15_Cl^−^, 943.5186). ^1^H-NMR (500 MHz, CDCl_3_) δ 5.69 (1H, d, *J =* 15.4 Hz, H-2), 6.99 (1H, dd, *J =* 15.3, 11.2 Hz, H-3), 6.14 (1H, dd, *J =* 15.1, 11.3 Hz, H-4), 5.64 (1H, dd, *J =* 15.0, 9.6 Hz, H-5), 2.54 (1H, m, H-6), 4.72 (1H, d, *J =* 10.3 Hz, H-7), 1.95 (1H, m, H-8), 4.10 (1H, m, H-9), 4.12 (1H, overlapped, 9-OH), 1.70 (1H, t, *J =* 7.5 Hz, H-10), 5.30 (1H, d, *J =* 2.0 Hz, 11-OH), 1.00 (1H, m, H-12a), 2.37 (1H, dd, *J =* 11.9, 4.5 Hz, H-12b), 3.95 (1H, m, H-13), 1.18 (1H, m, H-14), 3.91 (1H, m, H-15), 1.09 (3H, d, *J =* 6.2 Hz, H-16), 1.03 (3H, d, *J =* 6.6 Hz, H-17), 0.81 (3H, d, *J =* 6.8 Hz, H-18), 0.99 (3H, d, *J =* 7.2 Hz, H-19), 1.43 (1H, m, H-20a); 1.60 (1H, m, H-20b), 0.83 (3H, t, *J =* 7.5 Hz, H-21), 5.03 (1H, t, *J =* 2.1 Hz, H-22), 1.78 (2H, dd, *J =* 8.6, 2.4 Hz, H-23), 3.97 (1H, m, H-24), 3.60 (1H, br s, H-25), 3.98 (1H, m, H-26), 1.23 (3H, d, *J =* 6.6 Hz, H-27), 5.69 (1H, d, *J =* 15.4 Hz, H-2′), 6.99 (1H, dd, *J =* 15.3, 11.2 Hz, H-3′), 6.14 (1H, dd, *J =* 15.1, 11.3 Hz, H-4′), 5.64 (1H, dd, *J =* 15.0, 9.6 Hz, H-5′), 2.54 (1H, m, H-6′), 4.72 (1H, d, *J =* 10.3 Hz, H-7′), 1.95 (1H, m, H-8′), 4.10 (1H, m, H-9′), 4.12 (1H, overlapped, 9′-OH), 1.70 (1H, t, *J =* 7.5 Hz, H-10′), 5.23 (1H, d, *J =* 1.9 Hz, 11′-OH), 1.00 (1H, m, H-12′a), 2.45 (1H, dd, *J =* 12.0, 4.5 Hz, H-12′b), 3.47 (1H, td, *J =* 10.7, 4.7 Hz, H-13′), 1.13 (1H, m, H-14′), 3.85 (1H, m, H-15′), 1.08 (3H, d, *J =* 6.3 Hz, H-16′), 1.03 (3H, d, *J =* 6.6 Hz, H-17′), 0.81 (3H, d, *J =* 6.8 Hz, H-18′), 0.99 (3H, d, *J =* 7.2 Hz, H-19′), 1.43 (1H, m, H-20′a); 1.60 (1H, m, H-20′b), 0.86 (3H, t, *J =* 7.5 Hz, H-21′), 3.34 (3H, s, 13′-OCH_3_); ^13^C-NMR (125 MHz, CDCl_3_) δ170.3 (C-1), 170.2 (C-1′), 121.2(C-2, 2′), 145.3 (C-3, C-3′), 132.3 (C-4, C-4′), 144.5 (C-5, C-5′), 41.0 (C-6, C-6′), 78.1 (C-7), 77.4 (C-7′), 36.1 (C-8, C-8′), 70.9 (C-9), 70.8 (C-9′), 41.8 (C-10, C-10′), 99.3 (C-11), 99.4 (C-11′), 39.1 (C-12, C-12′), 70.4 (C-13), 76.1 (C-13′), 56.7 (13′-OCH3), 48.6 (C-14), 49.3 (C-14′), 66.7 (C-15), 67.3 (C-15′),19.5 (C-16), 19.4 (C-16′), 15.1 (C-17, C-17′), 8.9 (C-18, C-18′), 7.3 (C-19, C-19′), 19.6 (C-20, C-20′), 9.3 (C-21), 10.2 (C-21′), 93.4 (C-22), 33.8 (C-23), 66.1 (C-24), 71.7 (C-25), 66.3 (C-26), 17.0 (C-27).

### 3.4. Cytotoxic Activity Assay

Compounds **1**–**7** were evaluated for cytotoxic effects against HeLa and MCF-7 cells according to the previously described MTT method [29],. The absorbance at 570 nm was determined on a Tecan Infinite M200 Pro reader, and the reference wavelength was 690 nm. The experiment was conducted in triplicate. 5-Fluorouracil was employed as positive control.

## 4. Conclusions

Eight a group of elaiophylins comprised of a new derivative, halichoblelide D (**1**), together with seven known compounds **2**–**8** was isolated and identified from mangrove-derived *Streptomyces* sp. 219807. Compounds **1**–**7** displayed significant cytotoxic activities against HeLa and MCF-7 cells with IC_50_ values 0.19–2.12 μM, and were thus promising candidates for future development of antineoplastic drugs. Notably, the strain possesses the capacity of producing elaiophylin (**4**) reaching the hitherto highest yield (4486 mg/L) reported at lab scale.

## Figures and Tables

**Table 1:** ^13^C- and ^1^H-NMR data (500 MHz, CDCl_3_) for **1**.

Positions	δ_C_	δ_H_ (*J* in Hz)	Positions	δ_C_	δ_H_ (*J* in Hz)
1	170.3 s	-	1′	170.2 s	-
2	121.2 d	5.69 (d, 15.4)	2′	121.2 d	5.69 (d, 15.4)
3	145.3 d	6.99 (dd, 15.3, 11.2)	3′	145.3 d	6.99 (dd, 15.3, 11.2)
4	132.3 d	6.14 (dd, 15.1, 11.3)	4′	132.3 d	6.14 (dd, 15.1, 11.3)
5	144.5 d	5.64 (dd, 15.0, 9.6)	5′	144.5 d	5.64 (dd, 15.0, 9.6)
6	41.0 d	2.54 (m)	6′	41.0 d	2.54 (m)
7	78.1 d	4.72 (d, 10.3)	7′	77.4 d	4.72 (d, 10.3)
8	36.1 d	1.95 (m)	8′	36.1 d	1.95 (m)
9	70.9 d	4.10 (m)	9′	70.8 d	4.10 (m)
9-OH		4.12 (overlapped)			4.12 (overlapped)
10	41.8 d	1.70 (t, 7.5)	10′	41.8 d	1.70 (t, 7.5)
11	99.3 s	-	11′	99.4 d	-
11-OH		5.30 (d, 2.0)			5.23 (d, 1.9)
12	39.1 t	1.00 (m) 2.37 (dd, 11.9, 4.5)	12′	39.1 t	1.00 (m) 2.45 (dd, 12.0, 4.5)
13	70.4 d	3.95 (m)	13′	76.1 d	3.47 (td, 10.7, 4.7)
14	48.6 d	1.18 (m)	14′	49.3 d	1.13 (m)
15	66.7 d	3.91 (m)	15′	67.3 d	3.85 (m)
16	19.5 q	1.09 (d, 6.2)	16′	19.4 q	1.08 (d, 6.3)
17	15.1 q	1.03 (d, 6.6)	17′	15.1 q	1.03 (d, 6.6)
18	8.9 q	0.81 (d, 6.8)	18′	8.9 q	0.81 (d, 6.8)
19	7.3 q	0.99 (d, 7.2)	19′	7.3 q	0.99 (d, 7.2)
20	19.6 t	1.43 (m); 1.60 (m)	20′	19.6 t	1.43 (m); 1.60 (m)
21	9.3 q	0.83 (t, 7.5)	21′	10.2 q	0.86 (t, 7.5)
22	93.4 d	5.03 (t, 2.1)	13′-OCH_3_	56.7 q	3.34 (s)
23	33.8 t	1.78 (dd, 8.6, 2.4)			
24	66.1 d	3.97 (m)			
25	71.7 d	3.60 (br s)			
26	66.3 d	3.98 (m)			
27	17.0 q	1.23 (d, 6.6)			

**Table 2:** Cytotoxicities of compounds **1**–**7** against HeLa and MCF-7 cells.

Compounds	IC_50_ (μM)	
	HeLa	MCF-7
1	0.3	0.33
2	1.12	2.12
3	0.29	0.29
4	0.29	0.19
4–6 ^a^	0.57	0.96
7	0.59	0.79
5-Fluorouracil	770	770

^a^ Compounds **4**, **5** and **6** could partly interconvert when dissolved in MeOH [24].

## Supplementary Materials

The UV, IR, CD, NMR and HRMS spectra of compound **1**, and a standard curve of elaiophylin are available online at: http://www.mdpi.com/1420-3049/21/8/970.
