# Supplementary material for: Halichoblelide D, a New Elaiophylin Derivative with Potent Cytotoxic Activity from Mangrove-Derived Streptomyces sp. 219807
Source: Molecules. 2016 Jul 25;21(8):970. doi: 10.3390/molecules21080970 (PMC6273579; doi:10.3390/molecules21080970)
Supplement: Supplementary file 1 [file molecules-21-00970-s001.pdf]

Supplementary Materials: Halichoblelide D, a New Elaiophylin Derivative with Potent Cytotoxic Activity from Mangrove-Derived *Streptomyces* sp. 219807

Ying Han, Erli Tian, Dongbo Xu, Min Ma, Zixin Deng and Kui Hong

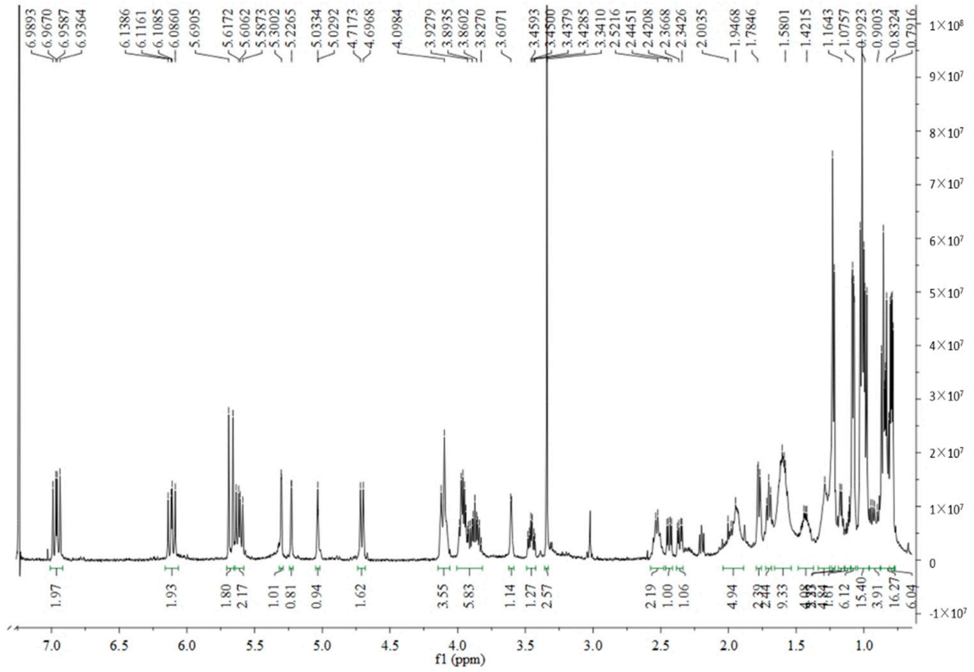

Figure S1. ^{1}H-NMR spectrum of **1** at 500 MHz in $CDCl_3$.

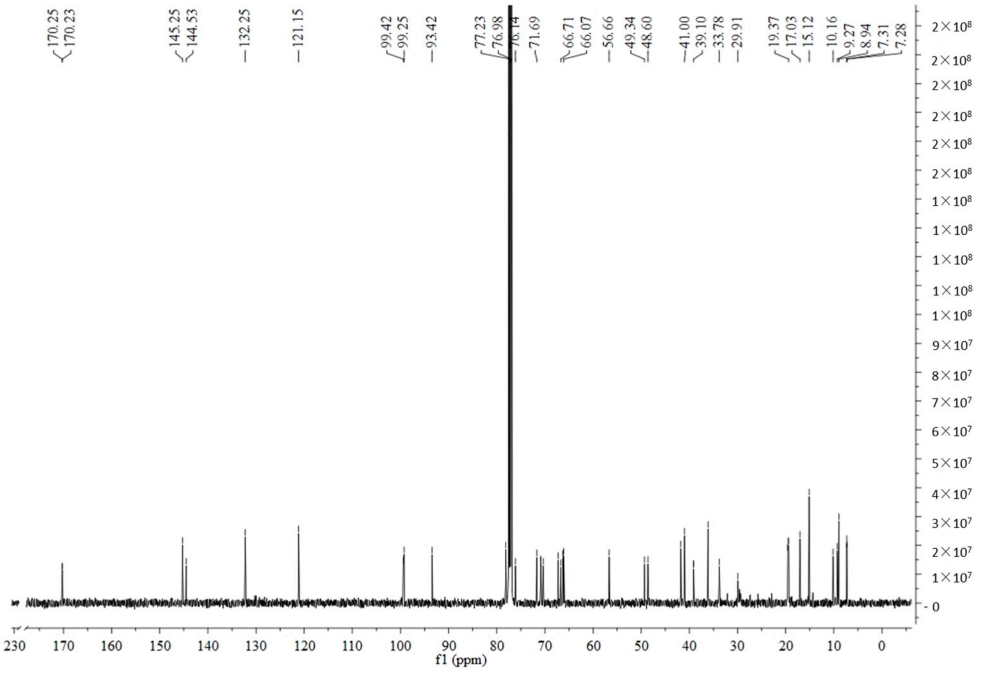

Figure S2. ^{13}C-NMR spectrum of **1** at 125 MHz in $CDCl_3$.

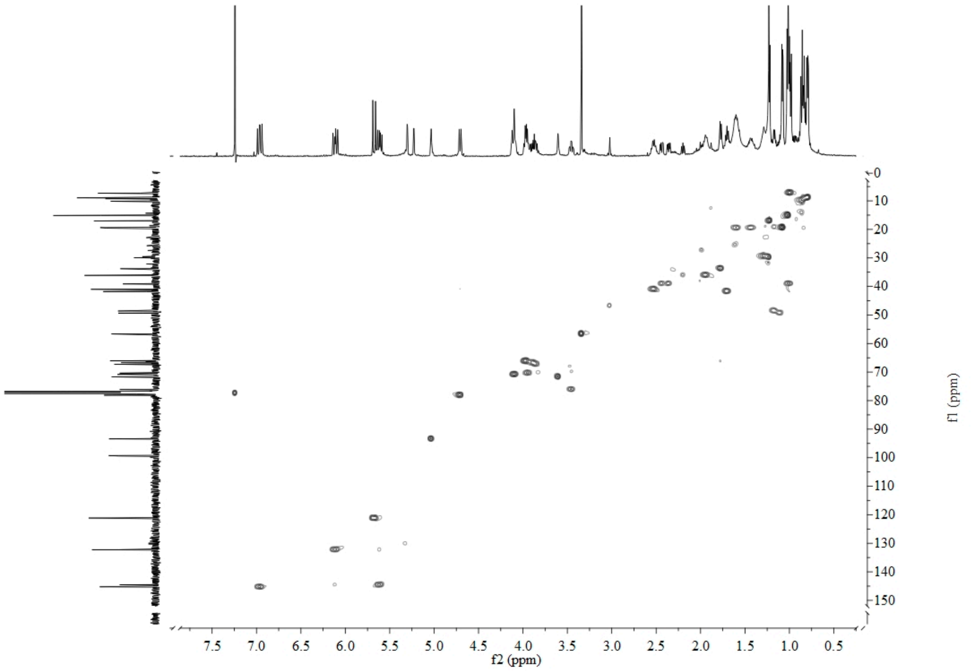

Figure S3. HSQC spectrum of **1** in $CDCl_3$.

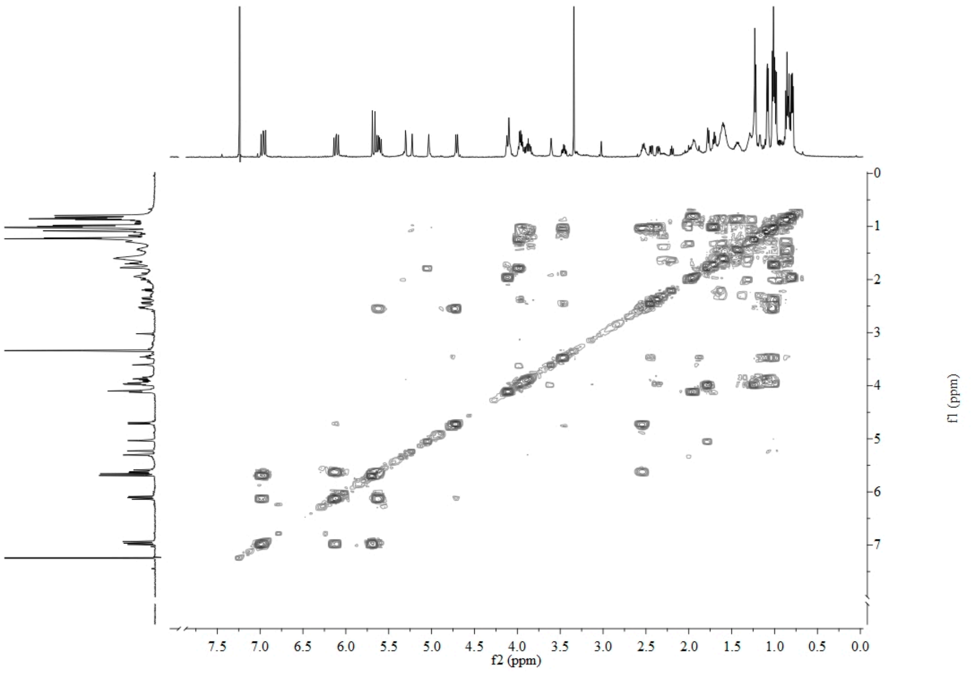

Figure S4. ^1H-^1H COSY spectrum of **1** in $CDCl_3$.

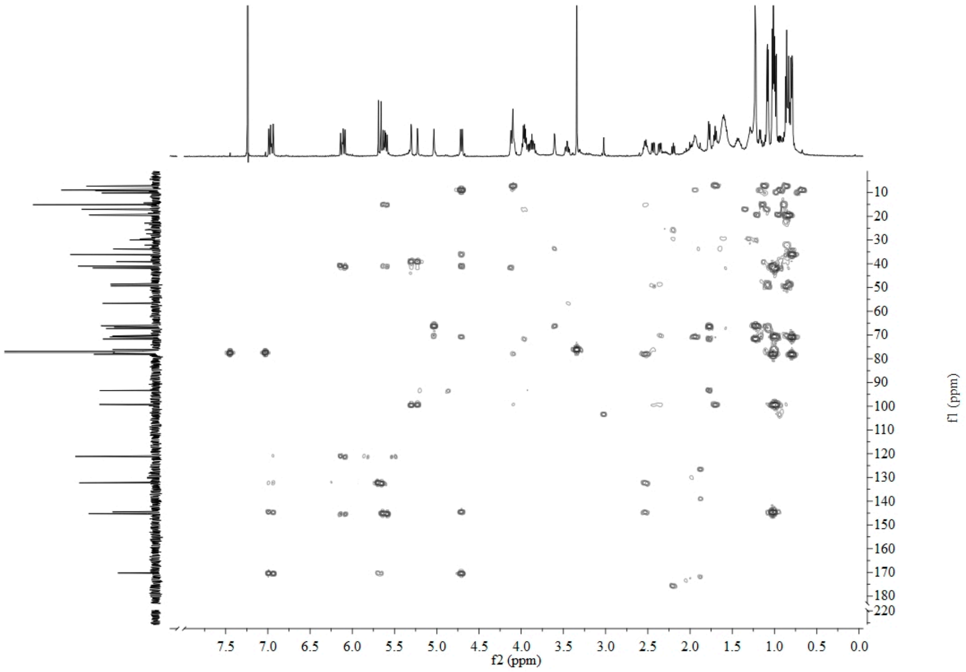

Figure S5. HMBC spectrum of **1** in $CDCl_3$.

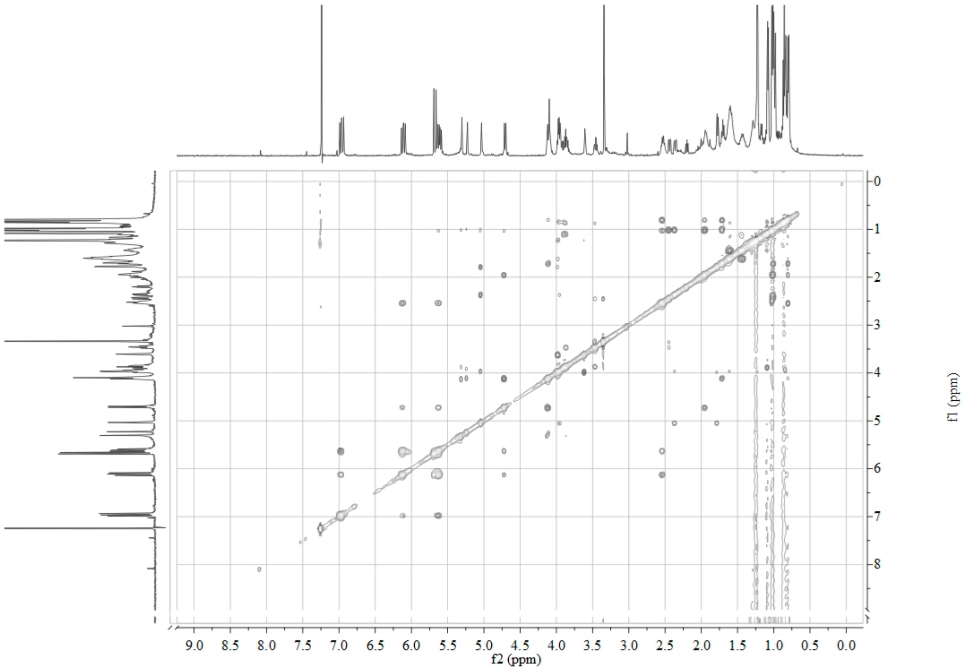

Figure S6. NOESY spectrum of **1** in $CDCl_3$.

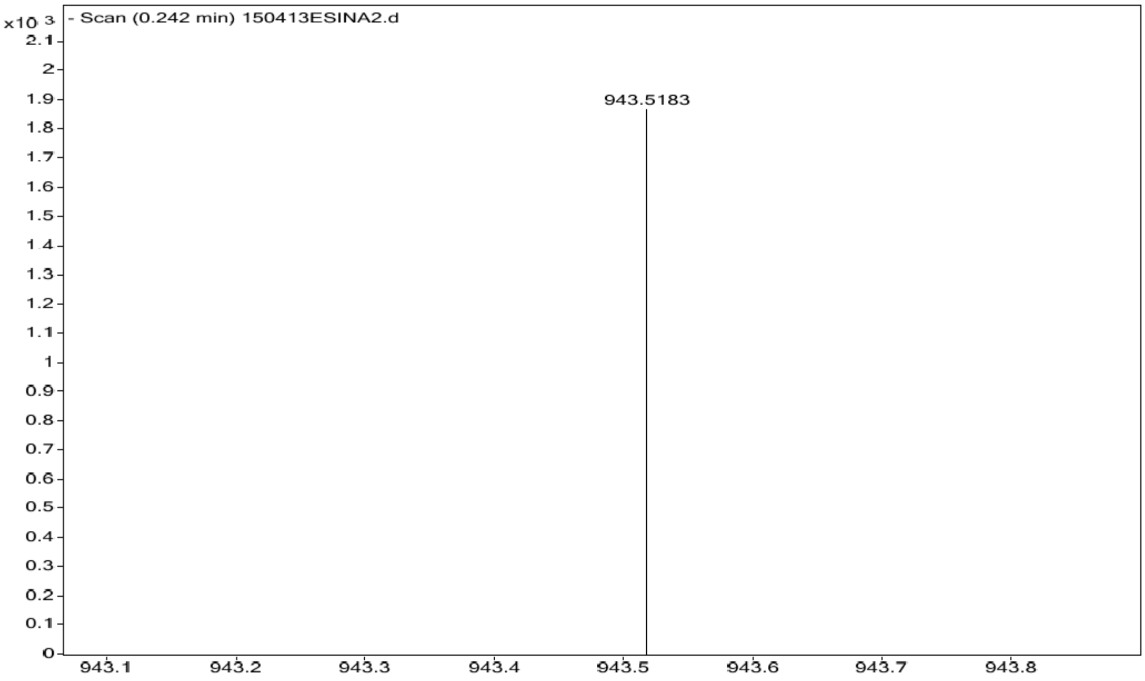

Figure S7. HRESIMS spectrum of **1**.

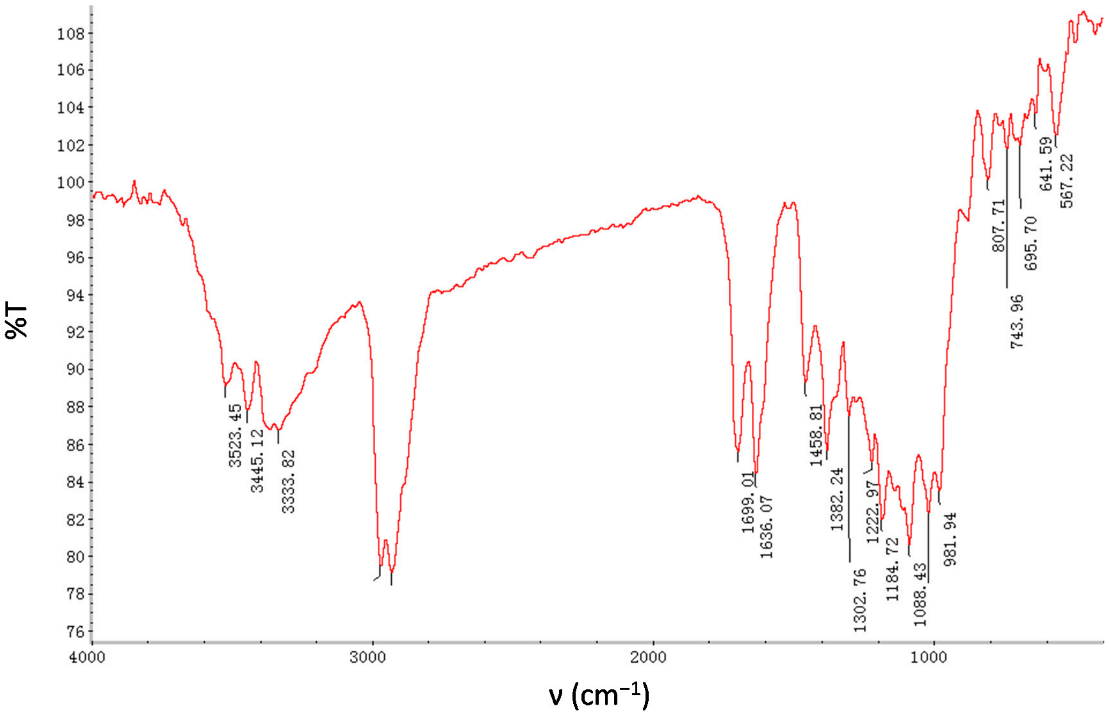

Figure S8. IR spectrum of **1**.

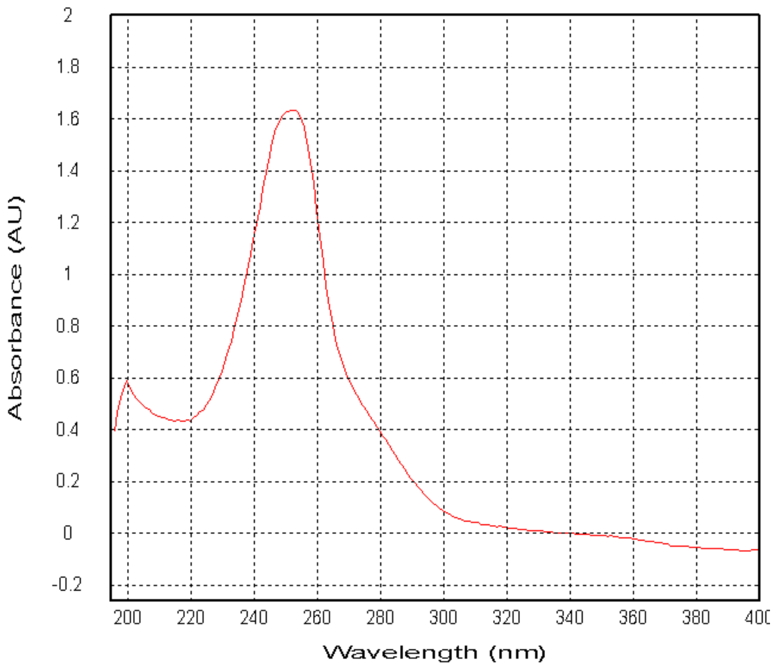

Figure S9. UV spectrum of **1**.

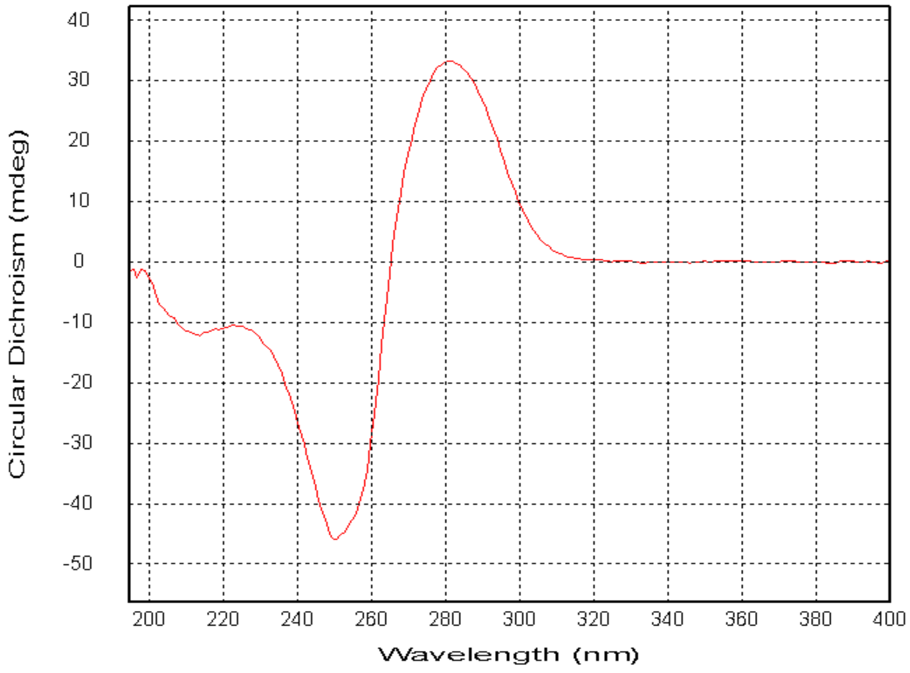

Figure S10. CD curves of compound **1** recorded in MeOH.

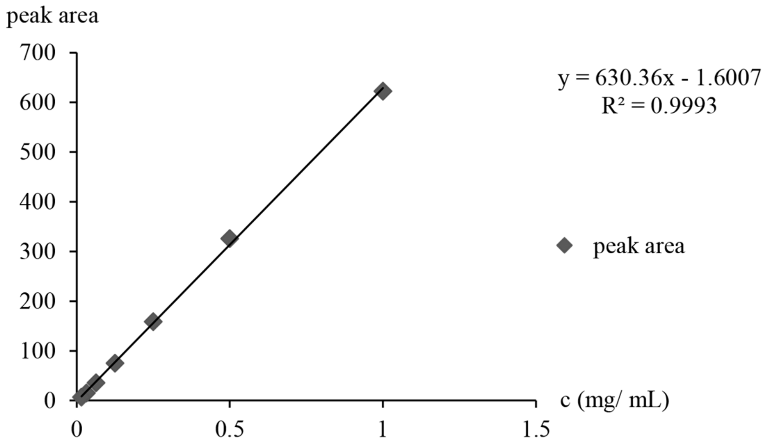

Figure S11. Standard curve of elaiophylin.

Table S1. ^{13}C-NMR (200 MHz) and ^{1}H-NMR (400 MHz) data of **4** in CD_3OD.

Position	δ_C	δ_H (Multi., *J* in Hz)
1	170.6 s	–
2	122.7 d	5.75 (d, 15.80)
3	147.0 d	6.93 (dd, 15.28, 11.16)
4	132. 9 d	6.16 (dd, 15.00, 11.20)
5	146.2 d	5.66 (dd, 15.04, 9.84)
6	42.9 d	2.58 (m)
7	78.4 d	5.01 (overlapped)
8	37.9 d	1.95 (m)
9	72.0 d	3.87 (m)
10	44.2 d	1.71 (m)
11	101.0 s	–
12	39.1 t	1.14 (m)<br>2.33 (dd, 12.28, 4.36)
13	71.1 d	3.91 (m)
14	50.0 d	1.27 (m)
15	68.3 d	3.88 (m)
16	19.7 q	1.12 (d, 6.08)
17	15.9 q	1.04 (d, 6.60)
18	9.8 q	0.86 (d, 6.64)
19	7.3 q	0.97 (d, 7.08)
20	20.5 t	1.46 (m); 1.67 (m)
21	9.7 q	0.87 (t, 7.2)
22	95.0 d	5.04 (overlapped)
23	33.9 t	1.62 (dd, 12.56, 4.64)<br>1.88 (m)
24	67.2 d	3.99 (d, 9.96)
25	72.5 d	3.52 (br s)
26	68.2 d	3.95 (m)
27	17.5 q	1.19 (d, 6.48)
